# Supplementary material for: Electron density modulation of NiCo2S4 nanowires by nitrogen incorporation for highly efficient hydrogen evolution catalysis
Source: Nat Commun. 2018 Apr 12;9:1425. doi: 10.1038/s41467-018-03858-w (PMC5897358; doi:10.1038/s41467-018-03858-w)
Supplement: Supplementary file 1 — Supplementary Information [file 41467_2018_3858_MOESM1_ESM.pdf]

Supplementary Information

Electron density modulation of $NiCo_2S_4$ nanowires by nitrogen incorporation for highly efficient hydrogen evolution catalysis

Yishang Wu[1,2#], *Xiaojing Liu*[1#], *Dongdong Han*[1], *Xianyin Song*[3], *Lei Shi*[1], *Yao Song*[1,2], *Shuwen Niu*[1], *Yufang Xie*[1], *Jinyan Cai*[1], *Shaoyang Wu*[1], *Jian Kang*[1], *Jianbin Zhou*[1], *Zhiyan Chen*[2], *Xusheng Zheng*[4]*, *Xiangheng Xiao*[3]*, *Gongming Wang*[1]*

[1]*Department of Chemistry, University of Science and Technology of China, Hefei, Anhui, 230026, P. R. China.*

[2]*School of Materials Science and Engineering, Central South University of Forestry and Technology, Changsha, 410004, P. R. China.*

[3]*Department of Physics, Wuhan University, Wuhan, 430072, P. R. China.*

[4]*National Synchrotron Radiation Laboratory, University of Science and Technology of China, Hefei, 230029, P. R. China.*

[#]*These authors contributed equally to this work.*

Corresponding Author

*Email: wanggm@ustc.edu.cn; zxs@ustc.edu.cn; xxh@whu.edu.cn.

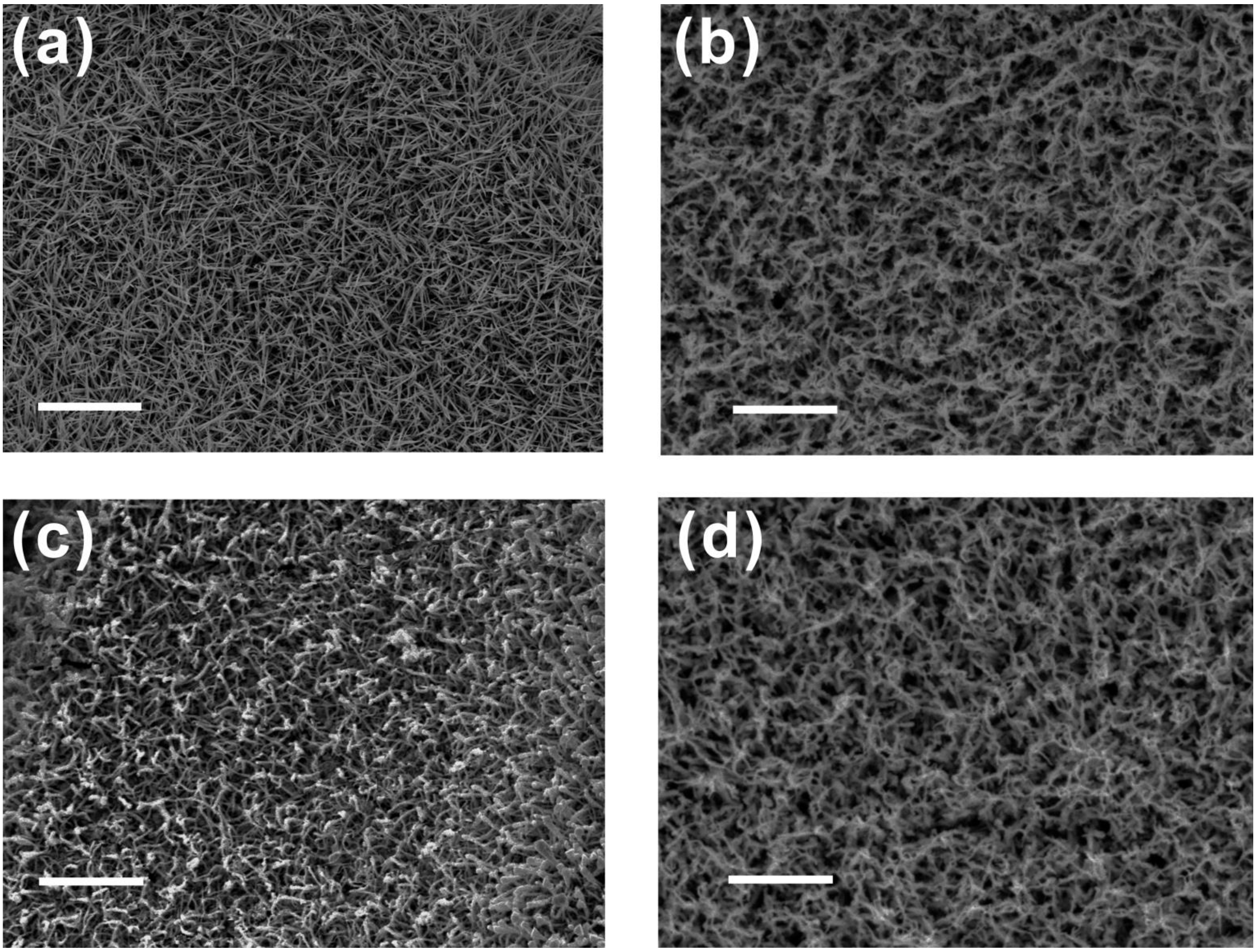

Supplementary Figure 1 | SEM images of (a) Ni-Co-O, (b) N-NiCoO, (c) $NiCo_2S_4$ and (d) N-$NiCo_2S_4$. Scale bars, 5 μm.

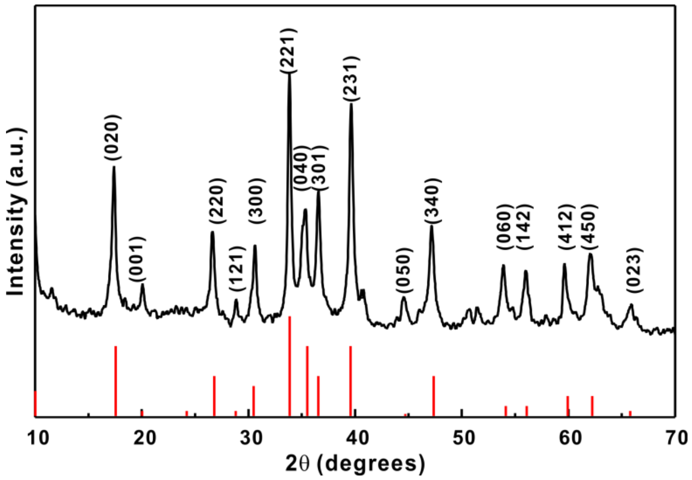

Supplementary Figure 2 | XRD pattern of Ni-Co-O NWs.

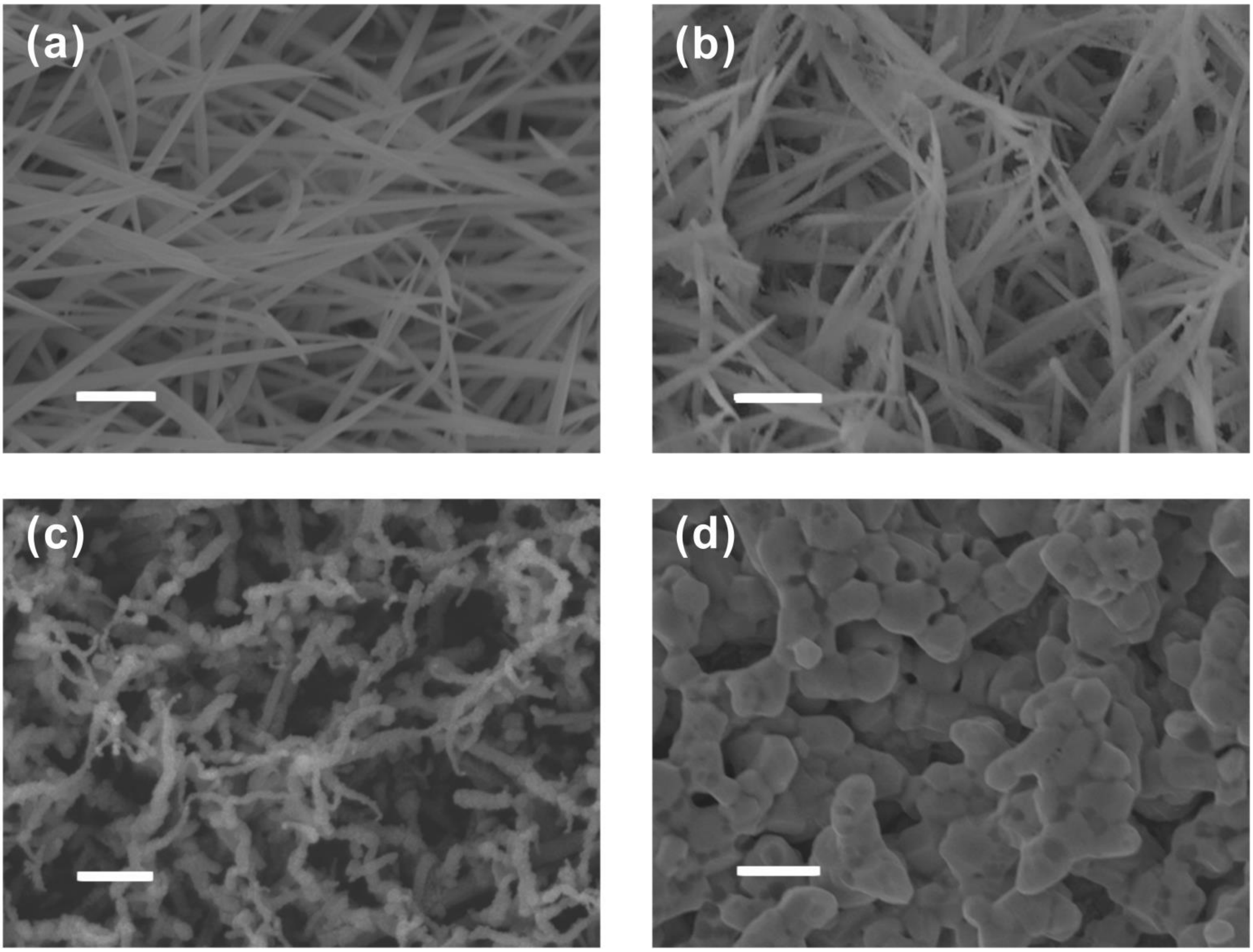

Supplementary Figure 3 | SEM images of N-$NiCo_2S_4$ prepared at different temperatures: (a) 400, (b) 500, (c) 600 and (d) 700 ^{o}C. Scale bars, 400 nm.

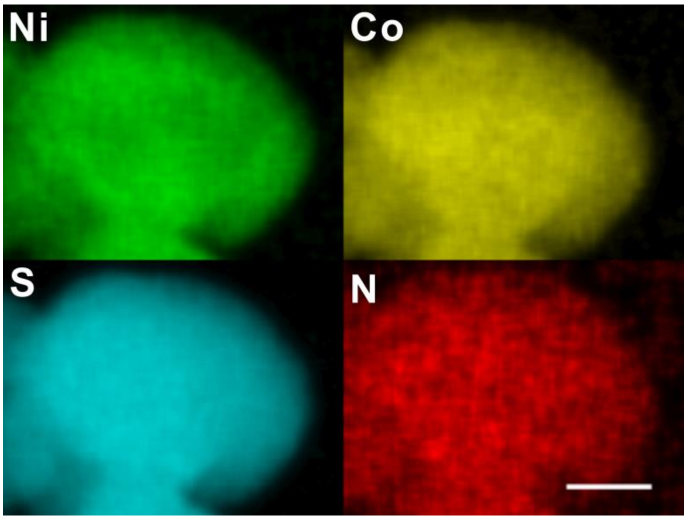

Supplementary Figure 4 | The element mapping images of Ni, Co, S and N elements in N-$NiCo_2S_4$ NW with a higher magnification. Scale bar is 10 nm.

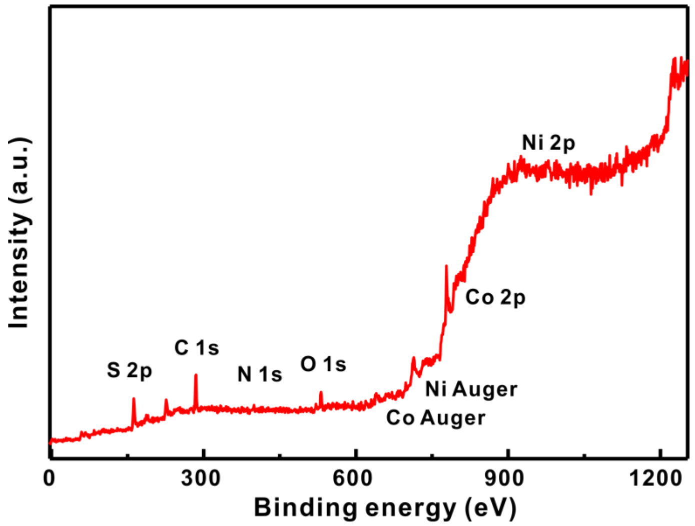

Supplementary Figure 5 | The XPS survey spectrum of N-$NiCo_2S_4$.

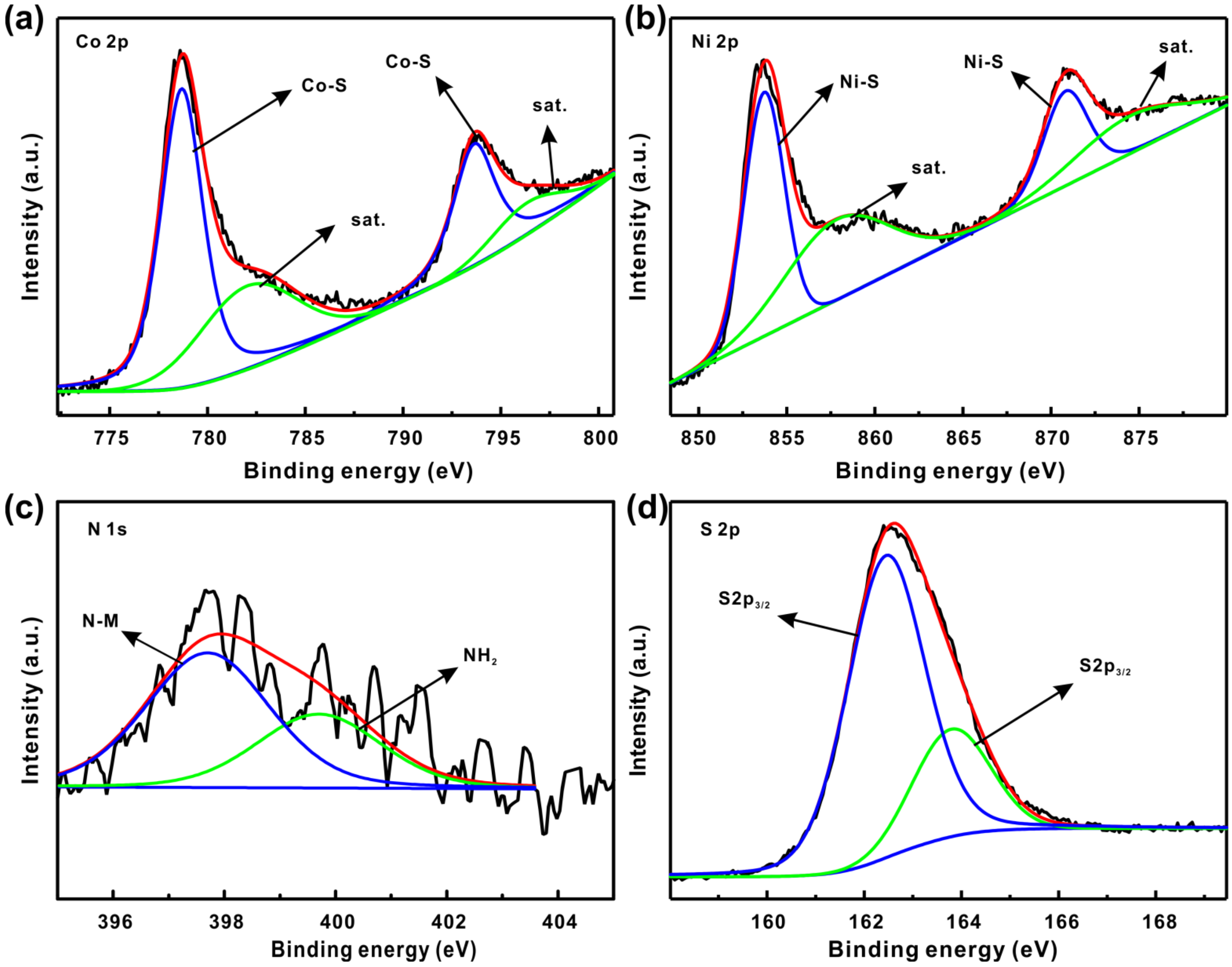

Supplementary Figure 6 | The deconvoluted core-level XPS spectra of (a) Co 2p, (b) Ni 2p, (c) N 1s and (d) S 2p in N-$NiCo_2S_4$ NWs.

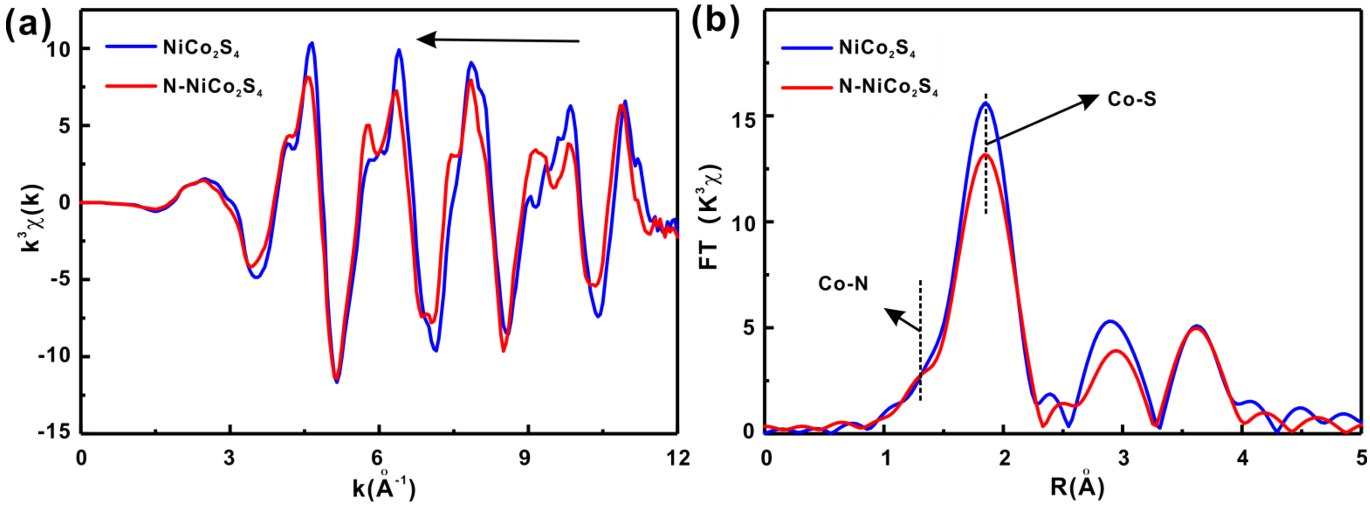

Supplementary Figure 7 | (a) k^3-weighted $\chi(k)$ spectra and (b) Fourier transformed Co K-edge extended XAFS spectra for $NiCo_2S_4$ (blue) and N- $NiCo_2S_4$ (red).

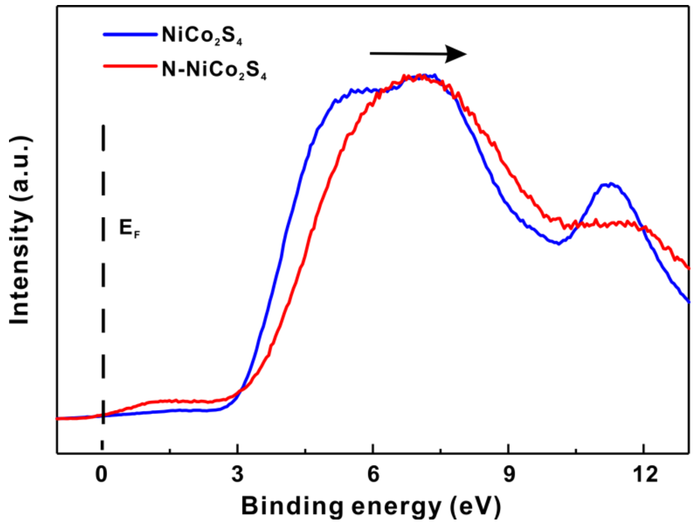

Supplementary Figure 8 | UPS VB spectra of $NiCo_2S_4$ (blue) and N-$NiCo_2S_4$ (red). The binding energy was calibrated by the E_F of Au foil.

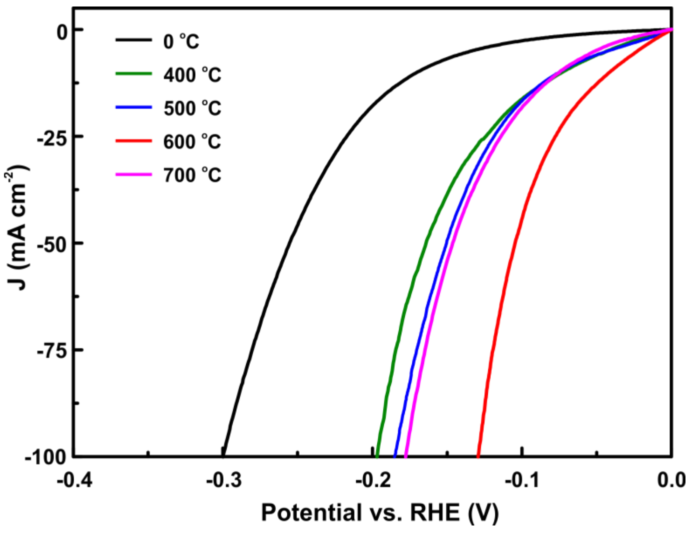

Supplementary Figure 9 | The LSV plots of N-$NiCo_2S_4$ prepared at different temperatures of 400, 500, 600 and 700 ^{o}C.

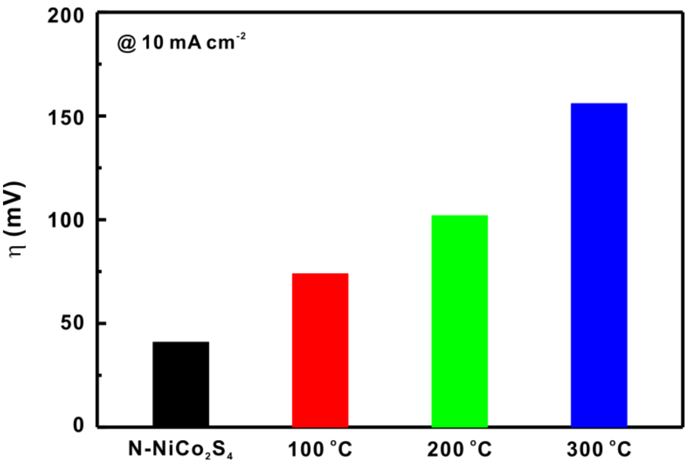

Supplementary Figure 10 | The overpotentials of N-$NiCo_2S_4$ NWs after being heated in air at different temperatures at the current density of 10 mA cm^{-2}.

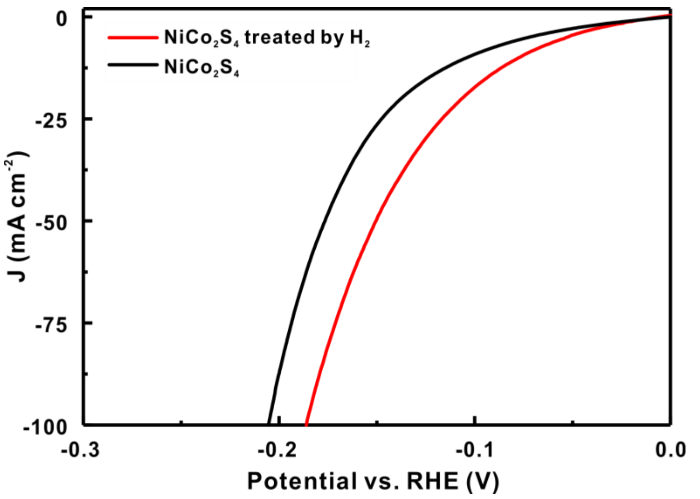

Supplementary Figure 11 | LSV curves of $NiCo_2S_4$ (black) and H_2 treated $NiCo_2S_4$ (red) at 300 °C.

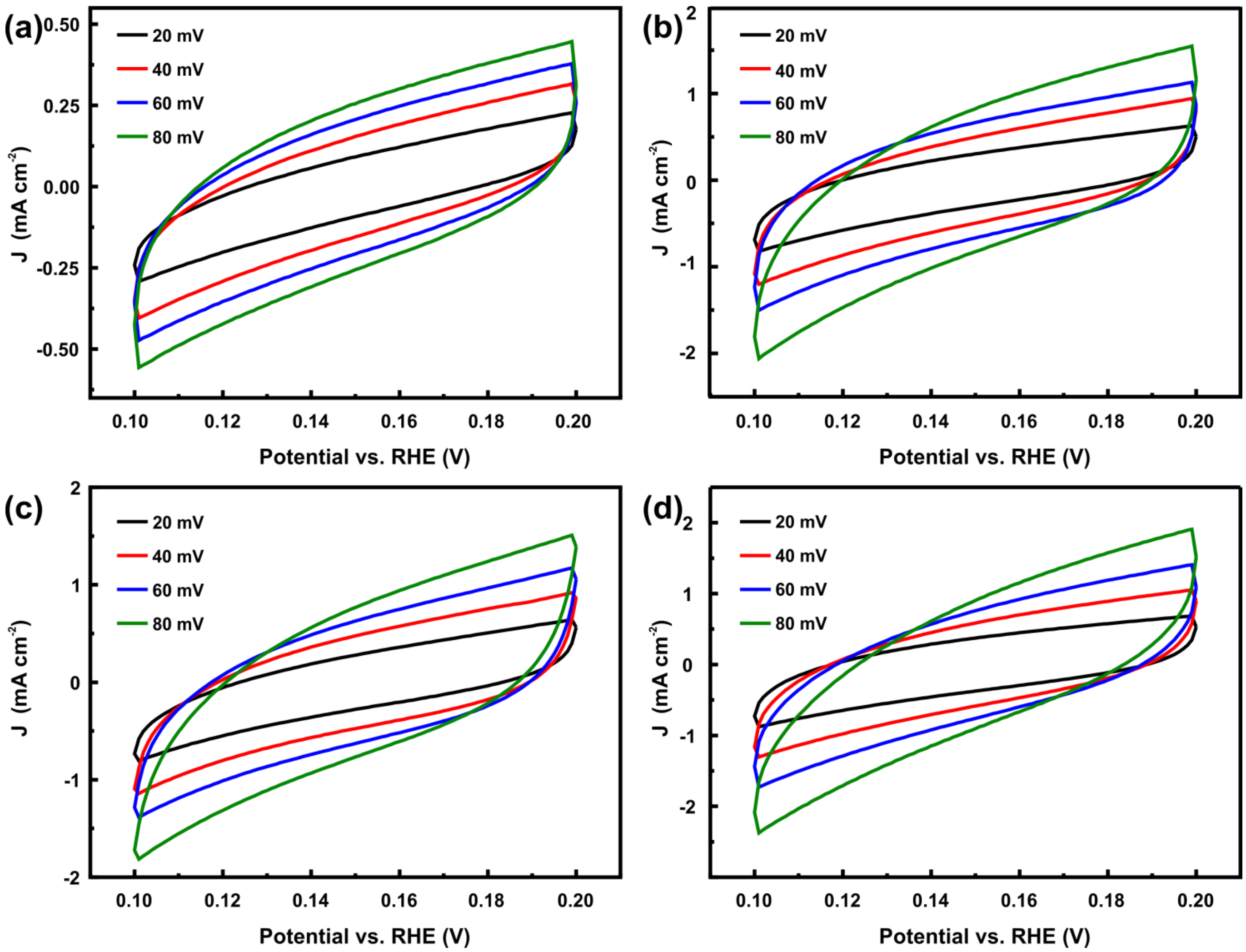

Supplementary Figure 12 | Cyclic voltammograms for (a) Ni-Co-O,(b) N-NiCoO (c) $NiCo_2S_4$, (d) N-$NiCo_2S_4$ NWs in the non-Faradaic capacitive range at the scan rates of 20 (black curve), 40 (red curve), 60 (blue curve) and 80 (cyan curve) mV

s^{-1}.

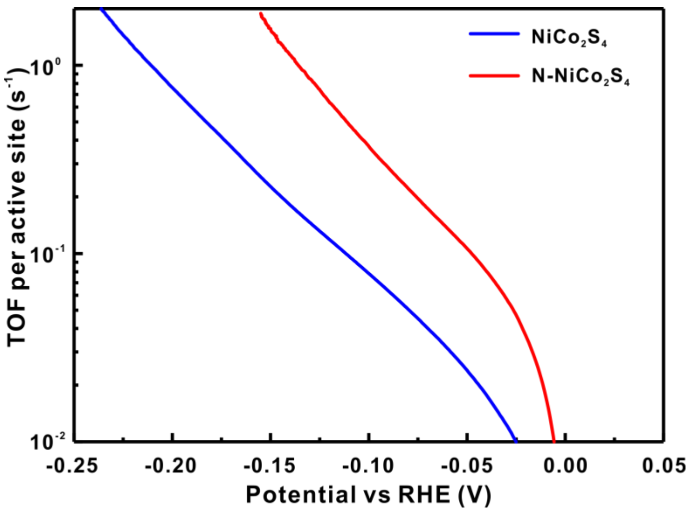

Supplementary Figure 13 | TOF against the potentials of $NiCo_2S_4$ (blue) and N-$NiCo_2S_4$ (red) for HER catalysis.

To calculate the TOF values, we used the previously reported calculation method[1-4]:

$$TOF=\frac{\text{number of total hydrogen turnovers / cm}^2\text{ of geometric area}}{\text{number of active sites / cm}^2\text{ of geometric area}}$$

The total number of hydrogen turnovers (No. of H_2) was obtained by the following equation.

$$\text{No. of } H_2 = (j\frac{mA}{cm^2})(\frac{1\ Cs^{-1}}{1000mA})(\frac{1\ mol\ e^-}{96485.3\ C})(\frac{1\ mol\ H_2}{2\ mol\ e^-})(\frac{6.022*10^{23}\ H_2\ molecules}{1\ mol\ H_2})$$

$$= 3.12*10^{15}\ \frac{H_2/s}{cm^2}\ \text{per}\frac{mA}{cm^2}$$

The number of active sites (No. of active sites) was estimated as the number of surface sites (including both Ni, Co and S atoms as the possible active sites). The active sites per real surface area is calculated from the following equation[3,5]:

$$\text{No. of active sites} = (\frac{\text{No. of atoms/unit cell}}{\text{volume/unit cell}})^{\frac{2}{3}}$$

The N-$NiCo_2S_4$ phase (JCPDS Card No.20-0782), a=b=c=9.329 (form DFT results), contains: 8 Ni, 16 Co, 31 S and 1 N atoms.

$$\text{No. of active sites (N-}NiCo_2S_4\text{)} = (\frac{56\ \text{atoms/unit cell}}{811.91\ \text{Å}^3\text{/unit cell}})^{\frac{2}{3}} = 1.68*10^{15}\ \text{atoms cm}^{-2};$$

$$\text{No. of active sites (}NiCo_2S_4\text{)} = (\frac{56\ \text{atoms/unit cell}}{825.03\ \text{Å}^3\text{/unit cell}})^{\frac{2}{3}} = 1.66*10^{15}\ \text{atoms cm}^{-2};$$

Finally, the plot of current density can be converted into a TOF plot according to the following formula[2]:

$$TOF = \frac{\left(3.12*10^{15}\frac{H_2/s}{cm^2}\text{per}\frac{mA}{cm^2}\right) * |J|}{\text{No. of active sites}*A_{ECSA}}$$

where the A_{ECSA} is electrochemical surface area (ECSA), which can be estimated using electrochemical double layer capacitance. The calculation equation is as follows[2].

$$A_{ECSA} = \frac{\text{specific capacitance}}{40\ \mu F\ cm^{-2}\ \text{per}\ cm^2_{ECSA}}$$

Where specific capacitance is C_{dl}; 40 μF cm^{-2} is reported for the calculated ECSA[2,6].

Clearly, the TOF values are significantly enhanced after nitrogen incorporation, suggesting nitrogen doping can intrinsically change catalytic activity of $NiCo_2S_4$.

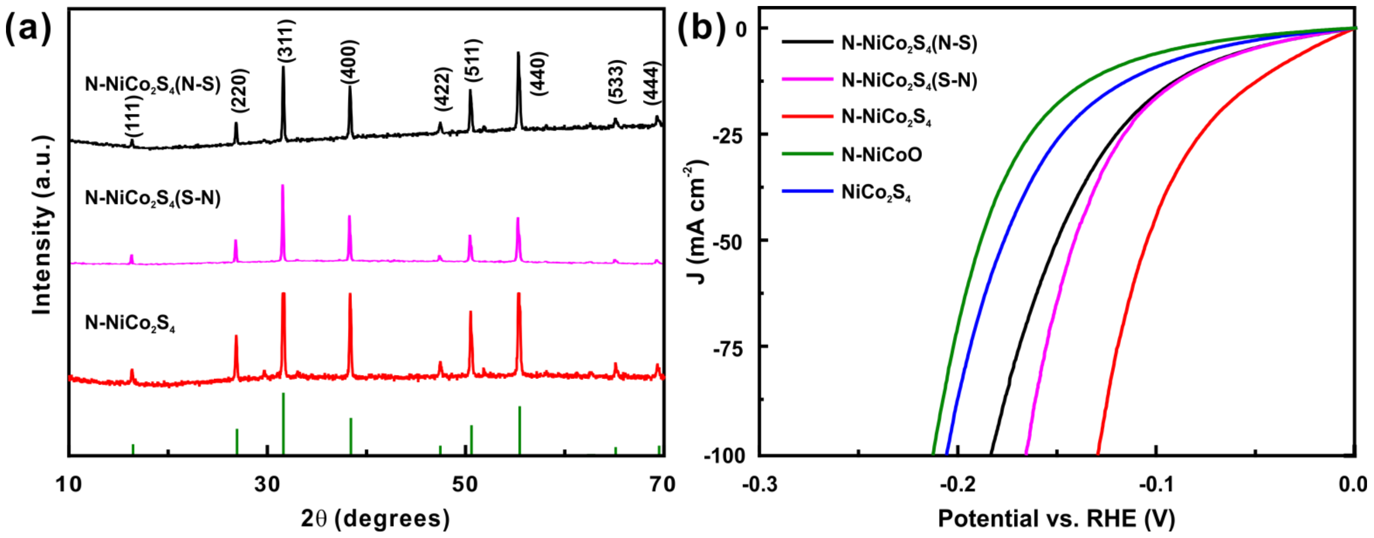

Supplementary Figure 14 | (a) XRD patterns of N-$NiCo_2S_4$ by pre-doping N into Ni-Co-O followed by sulfurization (N-S) and pre-sulfurization of Ni-Co-O followed by N doping (S-N). (b) The LSV profiles of N-$NiCo_2S_4$ prepared by different methods with a scan rate of 5 mV s^{-1} in 1.0 M KOH solution.

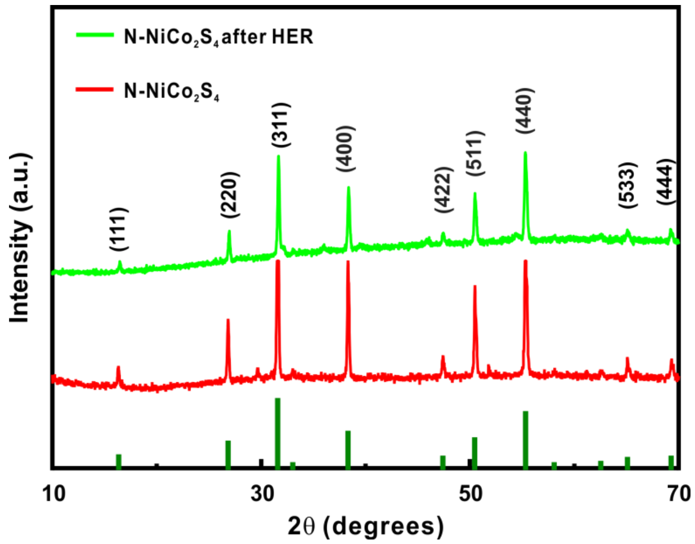

Supplementary Figure 15 | The XRD patterns of N-$NiCo_2S_4$ before and after stability test.

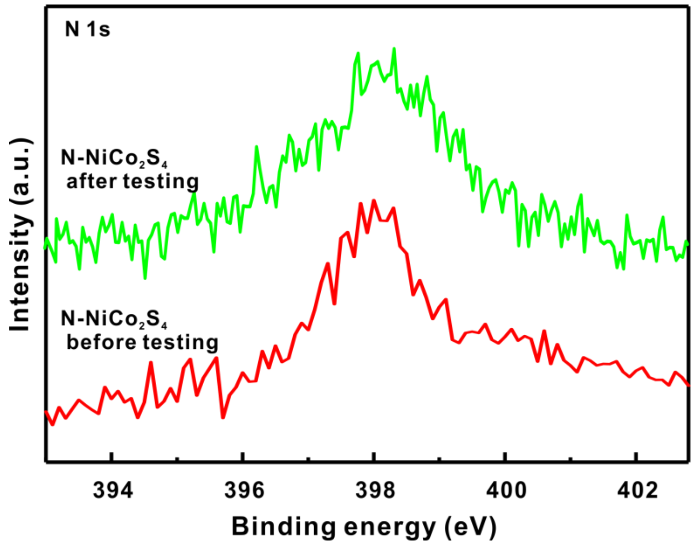

Supplementary Figure 16 | The XPS N 1s spectra of N-$NiCo_2S_4$ before and after stability test.

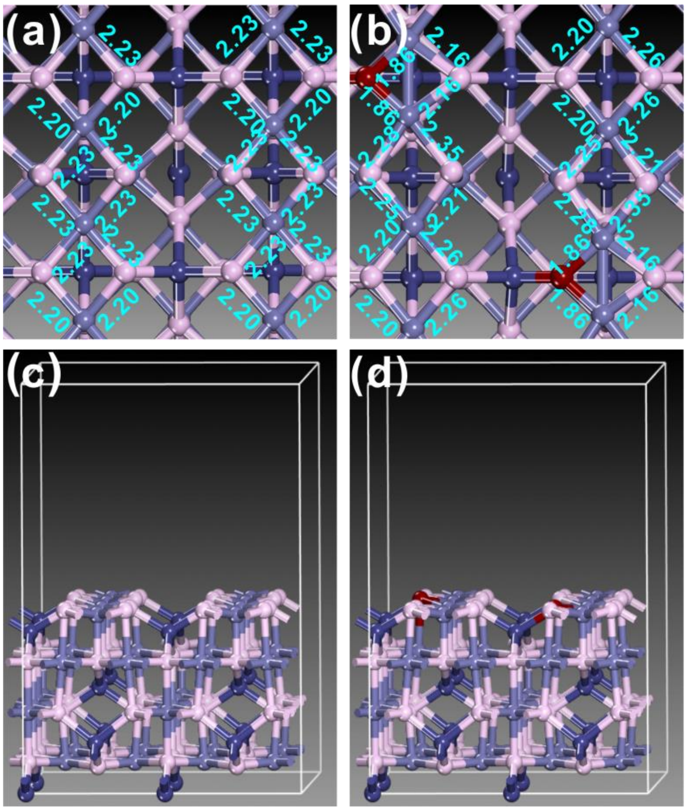

Supplementary Figure 17 | Top-view (a) (b) and side-view (c) (d) of the slab models of $NiCo_2S_4$ and N-$NiCo_2S_4$ (100) with labeled bond lengths.

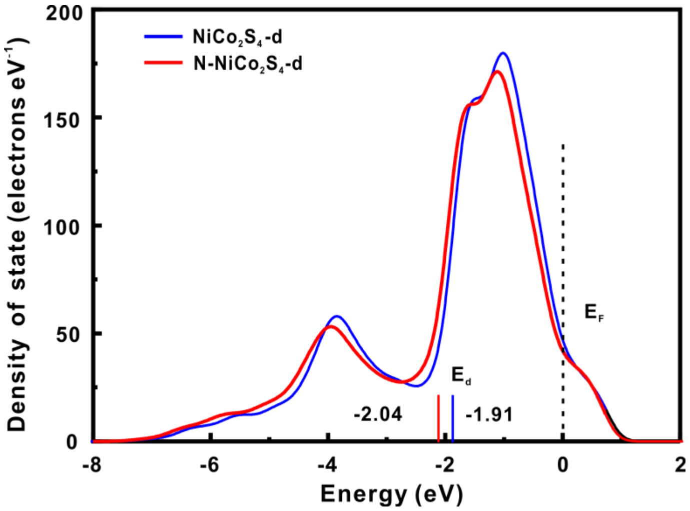

Supplementary Figure 18 | Density of state (DOS) plots of d orbital contribution to $NiCo_2S_4$ (blue) and N-$NiCo_2S_4$ (red). The dashed line is Fermi level.

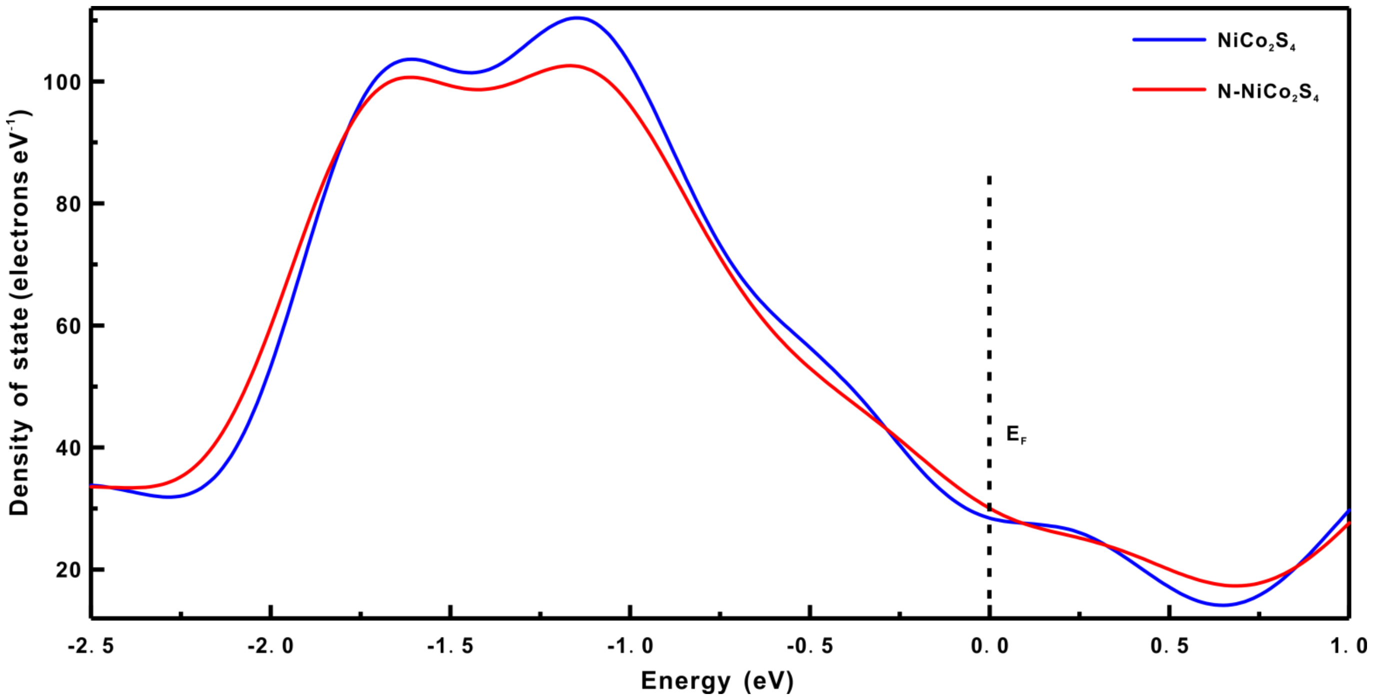

Supplementary Figure 19 | The density of state close to the Fermi level of bulk $NiCo_2S_4$ and bulk N-$NiCo_2S_4$.

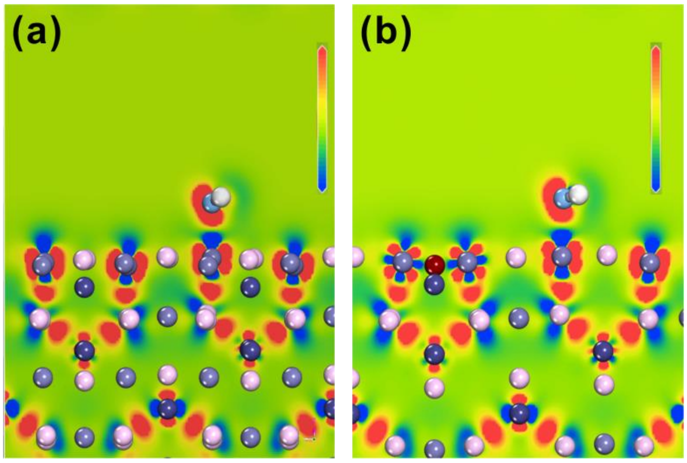

Supplementary Figure 20 | Side-view electron density difference images with adsorbed water on (a) $NiCo_2S_4$ and (b) N-$NiCo_2S_4$, ranging from -0.1 to 0.1.

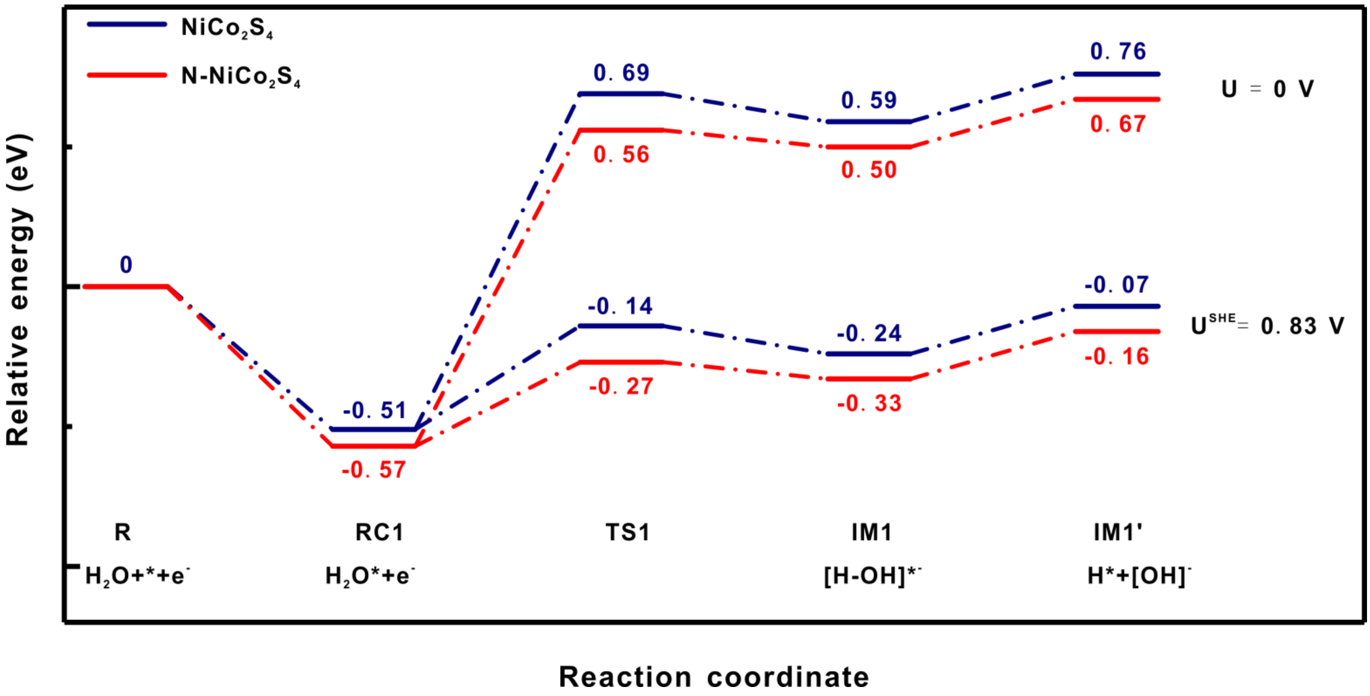

Supplementary Figure 21 | Relative energy profiles of the H* formation process on the Ni terminated surface of the $NiCo_2S_4$ (100) and N-$NiCo_2S_4$ (100) in alkaline medium at the electrode potentials of U = 0 V and U^{SHE} = 0.83V, respectively.

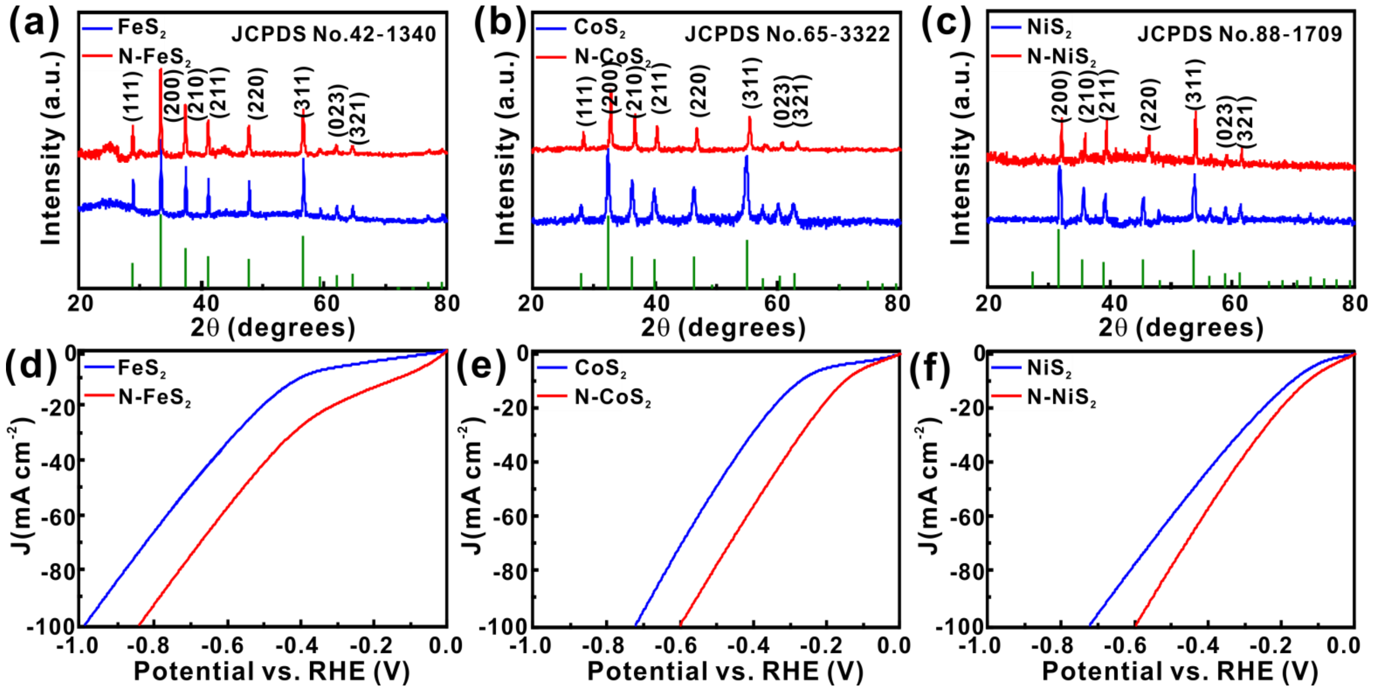

Supplementary Figure 22 | XRD patterns of (a) FeS_2 and N-FeS_2; (b) CoS_2 and N-CoS_2; (c) NiS_2 and N-NiS_2. The LSV plots of (d) FeS_2 and N-FeS_2; (e) CoS_2 and N-CoS_2; (f) NiS_2 and N-NiS_2 in 1.0 M KOH solution at the scan rate of 5 mV s^{-1}.

Supplementary Table 1 | The summary of representative metal sulfides-based HER catalysts.

Catalysts	η_{10} (mV)	Tafel slope (mV/ decade)	Electrolyte	Reference
Strained Vacancy-MoS_2	170	60	acidic	7
Exfoliated Metallic MoS_2	187	43	acidic	8
$NiCo_2S_4$	65	84.5	alkaline	9
N-doped and S Vacancy CoS_2	57	43	acidic	10
$NiMo_3S_4$	~257	98	alkaline	11
P-doped 2H-MoS_2	130	49	acidic	12
Cobalt-doped FeS_2	~100	~46	acidic	13
CoS	~162	93	acidic	14
$NiCo_2S_4$	210	58.9	alkaline	15
WS_2	~142	70	acidic	16
CoS_2	145	51.6	acidic	17
CoPS	48	56	acidic	18
Oxygen-Incorporated MoS_2	~160	55	acidic	19
Exfoliate WS_2	~221	55	acidic	20
**N-$NiCo_2S_4$**	**41**	**37**	**alkaline**	**This work**

Supplementary Table 2 | The collection of Tafel slope, $\eta_{10\ mA\ cm^{-2}}$, C_{dl}, normalized C_{dl}, J $_{(\eta=100mV)}$, and normalized $J_{(\eta=100mV)}$ of the Ni-Co-O, N-NiCoO, $NiCo_2S_4$, and N-$NiCo_2S_4$ NWs, respectively.

**Catalyst**	**Tafel slope (mV/ dec^{-1})**	$\eta_{10\ mA\ cm^{-2}}$ **(mV)**	C_{dl}	**Normalized** C_{dl}	**J** $_{(\eta=100\ mV)}$	**Normalized J** $_{(\eta=100\ mV)}$
Ni-Co-O	153	170	2.5	0.14	-2.5	-17.8
N-NiCoO	100	124	16	0.89	-6.0	-6.7
$NiCo_2S_4$	78	104	17	0.94	9.2	-9.8
N-$NiCo_2S_4$	37	41	18	1	-44.0	-44

References

1. Chen, Z.B. et al. Core-shell MoO_3-MoS_2 nanowires for hydrogen evolution: a functional design for electrocatalytic materials. *Nano Lett.* **11**, 4168-4175 (2011).
2. Zhang, R. et al. Ternary $NiCo_2P_x$ nanowires as pH-universal electrocatalysts for highly efficient hydrogen evolution reaction. *Adv. Mater.* **29**, (2017).
3. Popczun, E.J. et al. Nanostructured nickel phosphide as an electrocatalyst for the hydrogen evolution reaction. *J. Am. Chem. Soc.* **135**, 9267-9270 (2013).
4. Liang H. W. et al. Molecular metal-N_x centres in porous carbon for electrocatalytic hydrogen evolution. *Nat. Commun.* **6** 7992 (2015).
5. Laursen, A.B. et al. Nanocrystalline Ni_5P_4: a hydrogen evolution electrocatalyst of exceptional efficiency in both alkaline and acidic media. *Energy Environ. Sci.* **8**, 1027-1034 (2015).
6. Fang, M. et al. Hierarchical niMo-based 3D electrocatalysts for highly-efficient hydrogen evolution in alkaline conditions. *Nano Energy* **27**, 247-254 (2016).
7. Li, H. et al. Activating and optimizing MoS 2 basal planes for hydrogen evolution through the formation

of strained sulphur vacancies. *Nat. Mater.* **15**, 48-53 (2016).

8. Lukowski, M.A. et al. Enhanced hydrogen evolution catalysis from chemically exfoliated metallic MoS_2 nanosheets. *J. Am. Chem. Soc.* **135**, 10274-10277 (2013).

9. Ma, L. et al. Self-assembled ultrathin $NiCo_2S_4$ nanoflakes grown on Ni foam as high-performance flexible electrodes for hydrogen evolution reaction in alkaline solution. *Nano Energy* **24**, 139-147 (2016).

10. Zhang, J. et al. Activating and optimizing activity of CoS_2 for hydrogen evolution reaction through the synergic effect of N dopants and S vacancies. *Acs Energy Lett.* **2**, 1022-1028 (2017).

11. Jiang, J., Gao, M., Sheng, W. & Yan, Y. Hollow chevrel-phase $NiMo_3S_4$ for hydrogen evolution in alkaline electrolytes. *Angew. Chem. Int. Ed.* **55**, 15240-15245 (2016).

12. Huang, X. et al. Activating basal planes and S-terminated edges of MoS_2 toward more efficient hydrogen evolution. *Adv. Funct. Mater.* **27** (2017).

13. Wang, D.-Y. et al. Highly active and stable hybrid catalyst of cobalt-doped FeS_2 nanosheets–carbon nanotubes for hydrogen evolution reaction. *J. Am. Chem. Soc.* **137**, 1587-1592 (2015).

14. Sun, Y. et al. Electrodeposited cobalt-sulfide catalyst for electrochemical and photoelectrochemical hydrogen generation from water. *J. Am. Chem. Soc.* **135**, 17699-17702 (2013).

15. Sivanantham, A., Ganesan, P. & Shanmugam, S. Hierarchical $NiCo_2S_4$ nanowire arrays supported on Ni foam: an efficient and durable bifunctional electrocatalyst for oxygen and hydrogen evolution reactions. *Adv. Funct. Mater.* **26**, 4661-4672 (2016).

16. Lukowski, M.A. et al. Highly active hydrogen evolution catalysis from metallic WS_2 nanosheets. *Energy Environ. Sci.* **7**, 2608-2613 (2014).

17. Faber, M.S. et al. High-performance electrocatalysis using metallic cobalt pyrite (CoS_2) micro-and

nanostructures. *J. Am. Chem. Soc.* **136**, 10053-10061 (2014).

18. Cabán-Acevedo, M. et al. Efficient hydrogen evolution catalysis using ternary pyrite-type cobalt phosphosulphide. *Nat. Mater.* **14**, 1245 (2015).

19. Xie, J. et al. Correction to controllable disorder engineering in oxygen-incorporated MoS_2 ultrathin nanosheets for efficient hydrogen evolution. *J. Am. Chem. Soc.* **136**, 1680-1680 (2014).

20. Voiry, D. et al. Enhanced catalytic activity in strained chemically exfoliated WS_2 nanosheets for hydrogen evolution. *Nat. Mater.* **12**, 850 (2013).
